# Effectiveness of digital health interventions for increasing preventive care for smoking, nutrition, alcohol consumption, physical activity and weight (SNAP-W) in outpatient settings: a systematic review protocol

**DOI:** 10.1186/s13643-026-03123-y

**Published:** 2026-02-26

**Authors:** Eva Farragher, Carly Mallise, Emma Doherty, Simon R. E. Davidson, Rebecca Wyse, John Wiggers, Melanie Kingsland

**Affiliations:** 1 Hunter New England Population Health, Hunter New England Local Health District, Wallsend, New South Wales 2287 Australia; 2 School of Medicine and Public Health, The University of Newcastle, Callaghan, New South Wales 2308 Australia; 3 Hunter Medical Research Institute, New Lambton Heights, New South Wales 2305 Australia

**Keywords:** Digital health interventions, Preventive care, Smoking cessation, Nutrition, Alcohol, Physical activity, Weight, Outpatient, Primary care

## Abstract

**Background:**

Preventive care – asking, advising or referring patients for help with smoking, nutrition, alcohol, physical activity, and weight (SNAP-W) – is not consistently provided in routine outpatient care due to barriers such as time, other priorities, and forgetfulness. Digital health interventions (DHIs) integrated into routine care offer a promising solution and are acceptable to clinicians and patients. A systematic review is needed to synthesise existing evidence on the effectiveness of DHIs that engage patients, alongside routine care, to provide preventive care targeting SNAP-W in outpatient settings.

**Methods:**

We will include randomised and non-randomised studies that compare a DHI supporting the provision of preventive care for SNAP-W health behaviours with usual care. The DHI must integrate with routine clinician-provided care. Participants will be adult patients/clients of any outpatient healthcare service. The primary outcomes will be provision/receipt of preventive care elements addressing the SNAP-W health behaviours. Secondary outcomes will include SNAP-W behaviour change outcomes. Eligible studies will be identified via MEDLINE, EMBASE, PsycINFO, Scopus, and CINAHL. Two reviewers will independently conduct study selection, data extraction, and risk of bias assessment, with a third resolving disagreements. Risk of bias will be assessed using Cochrane RoB-2 for randomised and ROBINS-I for non-randomised trials. If feasible, a meta-analysis will be conducted to estimate the pooled effect of DHIs by health behaviour. Exploratory sub-group (e.g., type of clinical setting, preventive care element) analyses will be conducted to determine possible causes of statistical heterogeneity. If a meta-analysis is not feasible, results will be summarised using direction of effect per the Synthesis Without Meta-analysis guidelines.

**Discussion:**

This review will identify whether DHIs that engage patients as part of outpatient health care are effective at improving delivery of SNAP-W preventive care for adults attending these services. These findings will be of interest to service providers, policy makers and implementation researchers seeking to improve health outcomes through routine preventive care provision.

**Systematic review registration:**

PROSPERO CRD420251067831.

**Supplementary Information:**

The online version contains supplementary material available at 10.1186/s13643-026-03123-y.

## Background

Preventive care is a critical part of health systems worldwide. A key component of preventive care is addressing modifiable health behaviours including smoking/vaping, poor nutrition, alcohol misuse, physical inactivity, and overweight/obesity (SNAP-W) [1–4]. Globally, SNAP-W health behaviours are a major cause of morbidity and mortality and contribute to the development of chronic diseases, such as cardiovascular disease and cancer [5–7]. International guidelines recommend that preventive care related to SNAP-W health behaviours be delivered routinely in healthcare settings, including outpatient services such as maternity, mental health, community health, and primary care [8–12]. Multiple evidence-based models exist for the provision of preventive care, with the core elements being screening/assessment, brief advice, and referral or support. The 5As brief behavioural intervention framework [13, 14] (Ask, Advise, Assess, Assist, and Arrange), and the abbreviated versions Ask-Advise-Refer (AAR) and Ask-Advise-Help (AAH) [15, 16], are examples of these models.

Despite these recommendations, systematic reviews show that many patient groups receive sub-optimal and inconsistent levels of preventive care [17, 18]. Preventive care for the SNAP-W behaviours is inconsistently provided in mental health settings [19], with rates ranging from 13–100% for assessment, 16–100% for advice, and 5–92% for referrals. In primary care, there has been suboptimal provision of the 5As model for smoking [20] (ask: 65%, advise: 63%, assess: 36%, assist: 44%, arrange: 22%). Further, smoking cessation care during pregnancy [21] has been generally inconsistent with high rates for ask (91%) and advise (90%), but lower rates for assess (79%), assist (59%), and arrange (33%). Additionally, studies within maternity service settings [22–24] have found low levels of assessment (alcohol:14–64%, smoking: 79–96%, gestational weight gain (GWG): 13%), advice (alcohol: 9–35%, smoking: 62–73%, GWG: 30%), and referral to support services (alcohol: 0–50%, smoking: 38%, GWG: 10%). A study [25] of 1284 patients receiving community-based preventive care in Australia also found that less than two-thirds (60%) of clients received assessment for preventive health risks (smoking, alcohol use, inadequate fruit and vegetable consumption, or physical activity) and less than 5% received referral to support services.

Time, lack of priority and forgetting are the most cited barriers to the delivery of preventive care in clinical settings [26–29]. Preventive care provision is also affected by organisational constraints, such as lack of standardisation of processes and procedures [29]. Lack of staffing capacity, especially in rural or remote areas [28], has also been cited as a barrier to additional preventive care provision [27]. Solutions that address these barriers are needed if preventive care is to be delivered as intended as part of routine patient care across all service types and patient populations.

Digital health interventions (DHIs) offer a promising solution to address such barriers by delivering preventive care directly to patients [30]. DHIs have been shown to increase the provision of preventive health care to a range of patient groups [31, 32] and are reported to be acceptable and feasible by clinicians and consumers [33–35]. However, despite their potential, patient-facing DHIs often face sustainability and scale-up challenges due to lack of integration with routine health service delivery [31, 32, 36, 37]. As such, DHIs that integrate patient-facing technologies (e.g., online patient portals linked to electronic medical records, mobile health applications) into usual clinician-delivered care may be the most promising way to support sustained use and large-scale care improvement [38]. DHIs with such integrated functionality have been shown to improve patient access to their healthcare information, sharing of health information between patients and healthcare providers, and patient participation in care decisions [39]. As a result, DHIs have been found to improve patient health outcomes, such as blood pressure control and compliance with asthma management plans [40, 41]. Such care-integrated DHIs have great potential to support improvements to the delivery of preventive care (e.g., health assessments, brief advice, referrals) to patients [42]. A comprehensive evidence synthesis on the use of DHIs to support preventive care for patients attending healthcare settings is currently lacking and is required to determine their effectiveness and guide future research and practice considerations.

## Methods

### Aim

The aim of this systematic review is to examine the effectiveness of DHIs at increasing the provision of preventive care addressing SNAP-W health behaviours for adult patients in outpatient (i.e., non-admitted patient) healthcare settings, compared to usual care.

This systematic review has been registered with the International Prospective Register of Systematic Reviews (PROSPERO; registration number CRD420251067831). This review protocol was reported in accordance with the Preferred Reporting Items for Systematic Reviews and Meta-Analyses Protocol (PRISMA-P) recommendations for systematic review protocols [43] (see Additional file 1) and will be conducted using the Cochrane Handbook for Systematic Reviews of Interventions [44] for guidance. The review findings will be reported using the PRISMA guidelines [45].

### Eligibility criteria

#### Types of studies

Any of the following study designs that include a parallel comparison group will be eligible for inclusion: randomised study designs (including randomised controlled trials, randomised cluster studies, randomised staggered enrolment or stepped-wedge studies); non-randomised studies (including non-randomised controlled studies, non-randomised cluster studies, non-randomised staggered enrolment or stepped wedge studies); cohort studies; and mixed- and multi-method designs where the quantitative component includes a parallel comparison group. Studies which do not include a parallel comparison group, for example pre-post or case studies, will be excluded.

#### Participants

Participants will be adult patients/clients (aged 18 years or older) receiving clinical services from health professionals within outpatient (non-admitted) healthcare settings. This includes primary care (including general practice and family medicine), pre-surgery, specialist outpatient services, antenatal, prenatal, drug and alcohol, mental health, sexual health, oral health, pharmacies, specialist services, and diagnostic and assessment services (e.g., imaging centres). Health professionals providing this care may include nurses, medical doctors (e.g., clinicians, specialists), allied health workers, or other relevant health workers such as community or Aboriginal health workers. Studies that include patients/clients aged under 18 years, inpatient health settings, residential/aged care facilities, correctional facilities, or emergency/urgent care settings will be excluded.

#### Intervention

Studies that assess the effectiveness of a DHI in providing preventive care (ask, advise, refer or similar elements) targeting at least one of the SNAP-W health risks will be included. DHIs may support single or multiple preventive care elements for any SNAP-W risks, for example: alcohol consumption screening, dietary intake assessment, brief advice for smoking cessation, referral to physical activity. Further, the DHI needs to deliver preventive care directly to patients as well as be integrated with routinely delivered clinician care. The DHI could include digital technology including mobile phone texting, survey platforms, smartphone/tablet applications, web-based portals and modules, and data integration platforms (collecting information from various sources, e.g., medical records, wearables). Care provided to the patient via the digital tool must be available asynchronously (i.e., not in real-time). For example, an alcohol use assessment may be undertaken by patients via a digital tool prior to an appointment, with the clinician reviewing the assessment outcomes prior to a face-to-face appointment with the patient and follow up care. Studies will be excluded if the DHI: (a) is not integrated with routinely delivered clinician care (e.g., stand-alone apps); (b) includes a synchronously delivered digital strategy/tool, such as telehealth care (i.e., telephone/video call appointments), as the only digital strategy/tool; or (c) is solely clinician-facing (e.g., digital reminders for clinicians).

#### Comparison

Eligible studies will have a usual care comparison group where one or more elements of preventive care (ask, advise, refer or similar) addressing at least one SNAP-W behaviour is provided to patients by a clinician in either a face-to-face appointment or via synchronous telephone/video-based appointment.

### Outcomes

#### Primary outcome

The primary outcome will be any measure of provision/receipt of one or more preventive care components (assessment of risk, health advice, or linkage to further support) addressing at least one of the SNAP-W health behaviours. Commonly used terms and models include but are not limited to: ‘Ask-Advise-Refer [AAR]’, ‘Ask-Advise-Help [AAH]’, ‘5As [Ask-Advise-Assess-Assist-Arrange]’. Examples of these could be: alcohol use screening, education on healthy eating/dietary intake, provision of nicotine replacement therapy, or referral to smoking cessation support services. Measures of referral to support services can include offer, acceptance, or uptake measures. The provision of preventive care, delivered either through the DHI or by the clinician, can be measured using any quantitative data collection source, such as surveys (healthcare provider-reported or patient-reported), medical record audits, observations, or digital platform analytics.

#### Secondary outcomes

The secondary outcomes to be included are any behavioural change outcomes from the DHIs on any SNAP-W health behaviours or risks, which can be measured using any tool and reported in any format. Examples include tobacco smoking, use of e-cigarettes or vapes, alcohol consumption, dietary intake, minutes of physical activity undertaken per week, body weight, and body mass index.

### Other eligibility criteria

Studies may be published in any language, and there will be no date restrictions.

### Search strategy

The search strategy was developed in consultation with a research librarian who provided advice on the search sources.

#### Electronic sources

We will search MEDLINE, EMBASE via Ovid, PsycINFO, Scopus, and CINAHL (Cumulative Index to Nursing and Allied Health Literature) from database conception to present.

#### Other sources

Google Scholar will be used to perform a general hand search using the key search terms, which will be limited to the first 50 results as piloting the strategy showed low relevancy of results to the review beyond this point. Google Scholar will also be used to search for peer-reviewed original research articles based on relevant protocols, pre-prints, dissertations, and conference abstracts identified during the title and abstract screening phase. Reference lists of included articles in the review will be searched for other potentially eligible peer-reviewed original research articles. Peer-reviewed original research articles identified as relevant from the Google Scholar searches and reference lists will undergo title and abstract screening to determine eligibility for full-text review.

#### Search terms

The searches will include terms aligned with the eligibility criteria related to healthcare settings, digital care interventions, SNAP-W health behaviours, preventive care outcomes, and study designs. The search will be restricted to titles, abstracts, and key words and limited to journal articles. The proposed search strategy for the MEDLINE database is presented in Additional file 2. It will be adapted using appropriate syntax and terminology for other databases. The full search strategy from all databases will be published in the review paper.

### Data collection and analysis

#### Study selection

Covidence (web-based systematic review software) [46] will be used to facilitate the title and abstract screening and full-text review stages of the systematic review. Two reviewers will independently screen the de-duplicated titles and abstracts of all identified records for relevancy. Studies that are ineligible based on the review eligibility criteria from the initial title and abstract screen will be excluded. During full-text screening, two independent reviewers (who are blind to each other’s decision) will assess articles against the eligibility criteria. All studies will be screened/reviewed by at least one of the following research team members: CM, MK, ED, EF, or SD. Where there is insufficient information in the full text to determine eligibility, the study will be excluded from the review. Studies will be excluded according to a pragmatic, sequential order of exclusion criteria, and the reason for exclusion recorded for all full-text studies. Abstracts/full texts in languages other than English will be translated using Google Translate. Conflicts regarding study relevancy at the title and abstract screening stage and study eligibility at the full-text review stage will be resolved by a third reviewer (CM; or via consensus if conflict involves CM).

#### Data extraction

Two reviewers will independently extract information from eligible studies. A Research Electronic Data Capture (REDCap) [47] database will be used to facilitate data extraction. The review team will develop and pilot a double data entry form in REDCap that will allow for independent data extraction. Discrepancies in data extraction will be resolved by a third reviewer (CM). The following information will be extracted:

- Study characteristics: authors, date of publication, country of study, aim of study, study design.
- Participant characteristics: sample size, sex/gender, age, geographical location (e.g., urban, rural, regional), health condition(s), and equity characteristics pertinent to DHIs (e.g., socio-economic disadvantaged background, culturally or linguistically diverse background).
- Intervention characteristics: targeted SNAP-W health behaviour(s), targeted preventive care element(s), DHI description (technology type, core functionalities/features, integration/linkage to clinician-delivered care, DHI frameworks and co-design), health care setting, and/or provider profession.
- Comparison characteristics: Usual care description.
- Review primary and secondary outcomes: data collection method, effect sizes, and measures of outcome variability.

#### Assessment of risk of bias

The quality of the included studies will be assessed using RoB2 [48] for randomised controlled trials and ROBINS-I [49] for non-randomised trials. The review team will develop and pilot a double data entry form in REDCap that will allow for independent completion of the appraisal tools. Two reviewers will independently assess the quality of the included studies.

No studies will be excluded based on quality; however, the quality of the studies will be considered in interpreting the results using the Grading of Recommendations Assessment, Development and Evaluation (GRADE) system [50]. The GRADE quality ratings (from ‘very low’ to ‘high’) will be used to describe the body of evidence with randomised and non-randomised designs presented separately. Discrepancies regarding the quality appraisal will be resolved by consensus, or with a third reviewer with expertise in review methodology if agreement cannot be met.

#### Data synthesis

Study and participant characteristics will be summarised using descriptive statistics. The quality of the studies will be described using the results from the quality appraisal. Review primary outcomes will be synthesised separately, where possible, for each SNAP-W health behaviour (smoking/vaping, nutrition, alcohol consumption, physical activity, weight gain).

The primary analytical approach will be based on intention-to-treat (ITT) principles, with emphasis placed on reported effect estimates, where available, or between-group differences at follow-up rather than on changes from baseline. Review primary outcomes will be presented as odds ratios. Where study results are reported in other formats (e.g., means and standard deviations), these will be converted to odds ratios where possible to enable pooling in accordance with established methods [51, 52]. Where possible studies reporting multiple results for the same outcome (e.g., two measures of dietary intake) from the same sample of participants will be combined to create one summary effect for each outcome using recommended formulas. For secondary outcomes, the most suitable effect measure will be applied—odds ratios for dichotomous outcomes and means for continuous outcomes.

Where outcome measures from at least two studies can be pooled, meta-analyses (using either random or fixed effects models based on assessed homogeneity) will be conducted to estimate the overall pooled treatment effect for the primary outcomes, as well as by specific health behaviours for secondary outcomes (i.e., physical activity, alcohol consumption, dietary outcomes, and tobacco use). In cases where meta-analysis is not feasible due to insufficient or incomplete data (e.g., missing standard deviations that cannot be derived from reported data), results will be synthesised using direction of effect [48], following the Synthesis Without Meta-analysis (SWiM) guidelines [53].

For trials reporting multiple follow-up periods, data from the final follow-up will be used. Where multiple results are available for primary and secondary outcomes, preference will be given to the most objectively measured outcomes (e.g., analytics data over clinician self-report). Results from both cluster-randomised and individually randomised controlled trials will be combined. For cluster trials that do not account for clustering, standard errors will be adjusted for unit-of-analysis errors using procedures recommended in the Cochrane Handbook [48].

#### Dealing with missing data

The proportion of participants lost to follow-up will be documented and considered in the risk of bias assessment as a potential indicator of attrition bias. Data reported in accordance with the ITT principle will be prioritised for extraction. In instances where ITT data are not available, the best available data from the study will still be extracted and included in the synthesis.

#### Heterogeneity and subgroup analyses

If feasible, meta-analyses will be conducted on primary outcomes for studies with homogenous outcome measures (using *I*^*2*^ statistic to assess statistical heterogeneity). Study characteristics will be assessed to identify potential sources of intervention (e.g., preventive care element—assess, advise, refer or type of DHI) and methodological (e.g., study design—randomised, non-randomised trials) heterogeneity. Where necessary, forest plots will be visually inspected to evaluate statistical heterogeneity. If studies appear sufficiently homogeneous based on these preliminary assessments, statistical heterogeneity for each outcome will be quantified by calculating the* I*^*2*^ statistic, with values exceeding 50% considered indicative of substantial heterogeneity [44]. Decisions regarding the appropriateness of conducting meta-analyses will be made collaboratively among review authors, informed by these assessments of heterogeneity.

### Assessment of publication bias

Publication bias will be assessed using funnel plots if more than 10 studies are included in a meta-analysis. Asymmetry in the funnel plot will be evaluated visually and statistically using Egger’s regression test and Begg’s rank correlation test.

## Discussion

Modifiable health behaviours and associated risks, such as smoking/vaping, poor nutrition, alcohol misuse, physical inactivity, and overweight/obesity, are major contributors to the burden of non-communicable chronic diseases. Despite the existence of clinical guidelines worldwide, preventive care targeting these health behaviours is not routinely delivered in practice. Emerging evidence suggests that integrating DHIs into routine clinical care many be a solution. This systematic review will synthesise current evidence on the effectiveness of DHIs in improving the delivery of preventive care for SNAP-W health behaviours. The findings will be valuable to health services, researchers, and policymakers in supporting the development of DHIs that integrate into routine care to improve preventive health care delivery.

## Supplementary Information

Additional file 1. PRISMA-P 2015 Checklist.

Additional file 2. MEDLINE Search Term Strategy.

## Data Availability

Not applicable.

## Abbreviations

DHI: Digital health intervention
GWG: Gestational weight gain
GRADE: Grading of Recommendations Assessment, Development and Evaluation
ITT: Intention to treat
PRISMA-P: Preferred Reporting Items for Systematic Reviews and Meta-Analyses Protocol
PROSPERO: International Prospective Register of Systematic Reviews
RCTs: Randomised controlled trials
REDCap: Research Electronic Data Capture
SNAP-W: Smoking, nutrition, alcohol, physical activity, weight (health behaviours)
SWiM: Synthesis Without Meta-analysis
