# Supplementary material for: Effectiveness of digital health interventions for increasing preventive care for smoking, nutrition, alcohol consumption, physical activity and weight (SNAP-W) in outpatient settings: a systematic review protocol
Source: Syst Rev. 2026 Feb 26;15:129. doi: 10.1186/s13643-026-03123-y (PMC13069831; doi:10.1186/s13643-026-03123-y)
Supplement: Supplementary file 2 — Additional file 2. MEDLINE Search Term Strategy. [file 13643_2026_3123_MOESM2_ESM.docx]

**Effectiveness of digital health interventions for increasing preventive care for smoking, nutrition, alcohol consumption, physical activity and weight (SNAP-W) in outpatient settings: a systematic review protocol**

MEDLINE Search Term Strategy

((outpatient$1 or (health adj (service$1 or clinic$1 or facility or facilities or centre$1 or center$1 or practice$1)) or (ambulatory adj (service$1 or clinic$1 or facility or facilities or centre$1 or center$1 or practice$1)) or (medical adj (service$1 or clinic$1 or facility or facilities or centre$1 or center$1 or practice$1)) or (clinical adj (service$1 or practice$1)) or (medicine adj practice$1) or "primary care" or "primary health care" or "primary healthcare" or "general practice" or "family medicine" or (doctor's adj (surgery or surgeries)) or (antenatal adj1 (service$1 or clinic$1 or facility or facilities or centre$1 or center$1)) or

(prenatal adj1 (service$1 or clinic$1 or facility or facilities or centre$1 or center$1)) or (maternity adj1 (service$1 or clinic$1 or facility or facilities or centre$1 or center$1)) or (alcohol adj1 (service$1 or clinic$1 or facility or facilities or centre$1 or center$1)) or (drug adj1 (service$1 or clinic$1 or facility or facilities or centre$1 or center$1)) or (dental adj1 (service$1 or clinic$1 or facility or facilities or centre$1 or center$1 or practice$1)) or pharmacy or pharmacies or (imaging adj (service$1 or clinic$1 or facility or facilities or centre$1 or center$1)) or (diagnostic adj (service$1 or clinic$1 or facility or facilities or centre$1 or center$1)) or pre-surgery or pre-operation or pre-procedure) and (digital or electronic or e-health or platform or e-platform or portal or e-portal or dashboard

or e-visit or online or web-based or website or internet or computer or tablet or ((mobile or phone) adj application) or "mobile app" or "mobile phone" or "mobile device" or "smart phone" or m-health or "text message$1" or SMS or asynchronous* or store-and-forward or "information technolog*" or "information system$1") and (smok* or vaping or nutrition or "healthy eating" or diet or dietary or "fruit consumption" or "fruit intake" or "vegetable

consumption" or "vegetable intake" or "alcohol use" or "alcohol consumption" or "alcohol intake" or "alcohol drinking" or exercise or "physical activity" or (weight adj (loss or gain or management or maintenance or healthy or body)) or ("chronic disease" adj (risk$1 or factor$1)) or (lifestyle adj (risk$1 or factor$1 or behavio?r$1)) or (snap adj (risk$1 or factor$1 or behavio?r$1)) or (preventive adj (service$1 or care or health)) or (preventative adj (service$1 or care or health))) and ("AAR model" or "AAH model" or "5As" or advise* or advice or assess* or screen* or assist* or refer* or provide* or provision or received or

receipt or engage* or uptake or deliver* or counsel*) and ((randomi?ed adj5 (trial or group$1 or control* or "usual care" or intervention)) or (factorial adj (study or design)) or "quasi-experimental design" or "controlled before-and-a􀅌er" or "controlled pre-post" or (parallel adj1 (study or design or group or arm)) or "concurrent control group" or "concurrent comparison group")).ab,kf,ti. limited to Publication Types (Article).
